# The Adverse Effects of Thyrotropin Absence on Pancreatic *β* Cell Function in Mice

**DOI:** 10.1155/2019/9536032

**Published:** 2019-04-15

**Authors:** Yu Yang, Yu Chen, Jie Chen, Danyu Zhang, Jianhua Wang, Xiaodong Mao, Xiao Wei, Xingjia Li, Xianghua Ma, Chao Liu, Kun Wang

**Affiliations:** ^1^Department of Endocrinology, Affiliated Jiangning Hospital of Nanjing Medical University, Nanjing 211100, China; ^2^Department of Endocrinology, Affiliated Hospital on Integrated Traditional Chinese and Western Medicine, Nanjing University of Chinese Medicine, Nanjing 210023, China; ^3^Department of General Surgery, Affiliated Hospital on Integrated Traditional Chinese and Western Medicine, Nanjing University of Chinese Medicine, Nanjing 210023, China; ^4^Department of Endocrinology, The First Affiliated Hospital with Nanjing Medical University, Nanjing 210029, China

## Abstract

Thyrotropin (TSH) is a modulator of glucose metabolism by binding to its receptor on pancreatic cells. We used thyrotropin receptor (TSHR) knockout mice (*Tshr*^−/−^) as a model of TSH deletion to study its function in pancreatic *β* cells. *Tshr*^−/−^ mice had a similar body weight at birth compared with *Tshr*^+/+^ mice, but grew at a significantly slower rate until adulthood with adequate thyroxine supplementation. TSH deletion led to lower fasting and postprandial blood glucose, insulin secretion impairment, and atrophy of islets in adult mice. Transcription factors and markers of pancreatic *β* cell maturation, Pdx1, Nkx6.1, Glut2, and insulin, together with cell proliferation marker Ki67 showed no differences at the mRNA level between the two groups. However, the Bax/Bcl-2 ratio was remarkably elevated in *Tshr*^−/−^ mice at both mRNA and protein levels. We hypothesized that pancreatic cell apoptosis, rather than abnormal cell proliferation and maturation, is associated with pancreatic dysfunction and glucose intolerance in the absence of TSH modulation.

## 1. Introduction

The extrathyroidal effect of thyrotropin (TSH) has raised concerns in recent years. An abnormal TSH level can disturb the metabolic status in adipose tissue, bone, gonads, and the immune system [1]. Recently, its role in regulating glucose metabolism has attracted attention. It has been reported that an elevated TSH level is related to impaired glucose metabolism [2, 3] and poor glycemic control in type 2 diabetic patients [4]. Bilic-Komarica et al. found that normalization of TSH after levothyroxine treatment decreases fasting insulin and fasting and postprandial glucose in patients with subclinical hypothyroidism [5]. A large scale epidemiological investigation also revealed that a high TSH level is associated with increased fasting glucose and insulin resistance in a euthyroid population [6, 7]. These studies suggest that TSH may participate in glucose metabolism.

TSH acts as a metabolic regulator after binding to TSH receptor (TSHR) that has been detected in various tissues, including the brain, kidneys, heart, liver [8], and pancreatic islets [9]. Decreased fasting blood glucose is observed in TSHR knockout mice, which is ascribed to decreased hepatic glucose production [8], and TSHR-Asp727Glu polymorphism is associated with insulin resistance in nondiabetic older men [10]. Glucose homeostasis results from the balance of production and utilization of glucose. Pancreatic islets are the major organ that participates in balancing blood glucose, and the normal functions of *β* cells ensure sufficient insulin secretion. It has been reported that TSH stimulates insulin secretion in INS-1 cells by upregulating expression of glucose transporter 2 (Glut2) [1]. However, little is known about whether TSH blockade affects the functions of pancreatic *β* cells, the central region of glucose metabolism. We hypothesized that TSHR expressed on pancreatic *β* cells allows TSH to act as a regulator to maintain normal *β* cell functions and blood glucose homeostasis. In our study, TSHR knockout (*Tshr*^−/−^) mice were used to investigate whether the absence of TSH regulation affected the functions of pancreatic *β* cells.

## 2. Materials and Methods

### 2.1. Chemicals

Bovine serum albumin (BSA), Hank's balanced salt solution (HBSS), RPMI 1640 medium, and collagenase V were obtained from Sigma-Aldrich (St. Louis, USA). Fetal bovine serum (FBS) was purchased from Gibco (Grand Island, USA). A mouse anti-insulin antibody was purchased from Abcam (Cambridge, MA). Rabbit anti-Bax, rabbit anti-Bcl-2, rabbit anti-caspase-3, and rabbit anti-GAPDH antibodies as well as secondary antibodies were obtained from Cell Signaling Technology (Beverly, USA). 2-(4-Amidinophenyl)-6-indolecarbamidine dihydrochloride (DAPI) was purchased from Beyotime Institute of Biotechnology (Nantong, China).

### 2.2. Animals and Treatment

Eight-week-old female (*n* = 6) and male (*n* = 3) heterozygous (*Tshr*^+/-^) mice were donated by Prof. Jiajun Zhao, Provincial Hospital Affiliated to Shandong University (Jinan, China) and housed in the Animal Research Center of the Affiliated Hospital on Integrated Traditional Chinese and Western Medicine, Nanjing University of Chinese Medicine. Food and water were provided ad libitum. Mice were kept in a 12 h : 12 h light : dark cycle in a temperature-controlled room (22 ± 2°C). After 4 weeks of acclimation, heterozygous mice were bred to obtain *Tshr*^+/+^ and *Tshr*^−/−^ mice. Genotyping of the offspring was performed at 3 weeks of age. *Tshr*^−/−^ offspring were fed chow containing 100 ppm L-thyroxine powder (Sigma-Aldrich, USA).

The care and treatment of mice were conducted in accordance with the recommendations in the Guide for the Care and Use of Laboratory Animals of the National Institutes of Health. This study complied with the current Chinese laws pertaining to the conduct of scientific research.

### 2.3. Determination of Gene Expression in Male Offspring

DNA of male offspring from tail tips was extracted according to the manufacturer's instructions (DNeasy Blood & Tissue Kit, Qiagen, USA). The purity of DNA was determined by the OD at 260/280 nm. Primer sequences are shown in Table 1. Amplification was carried out in a reaction mixture including 2 *μ*L of 10 × buffer, 2 *μ*L dNTP, 1.5 *μ*L MgCl_2_, 0.1 *μ*L Taq polymerase, 2 *μ*L DNA product, 0.4 *μ*L forward and reverse primers, and 11.6 *μ*L ddH_2_O. The PCR product (10 *μ*L) mixed with SYBR was analyzed using a 1% agarose gel and observed under UV light. The bands of TSHR in wildtype and mutant mice were located at 590 and 173 kb, respectively.

Total RNA was extracted from the pancreas of male offspring using TRIzol Reagent (Thermo Fisher Scientific, USA). Primers were dissolved in water to the recommended concentration according to general data sheets (10 nmol/mL). A first-strand cDNA synthesis kit (TOYOBO, Japan) was used for reverse transcription. Quantitative real-time PCR was performed with the QuantStudio Dx System (Life Technology, USA) using the SYBR Green method. PCR cycling was 95°C for 1 min and then 40 cycles of 95°C for 15 sec, 55°C for 15 sec, and 72°C for 45 sec. Each sample was run in triplicate and normalized to GAPDH expression. Relative quantification values were determined using the 2^−ΔΔCt^ method. Primer sequences are shown in Table 1.

### 2.4. Body and Pancreas Weights

Body weight was measured at birth as well as weaning and adulthood (3 and 8 weeks old, respectively). The pancreas was excised and weighted at 8 weeks old, and then frozen at −80°C until use.

### 2.5. Serum Hormone Assay

Blood samples (200–300 *μ*L) were collected from the fossa orbitalis and stored at 4°C overnight. Then, the samples were centrifuged at 3000 rpm for 20 min, and the sera were collected (50–100 *μ*L). Serum T4 and TSH were measured by ELISA kits (Cloud-Clone Corp., USA). The detection ranges of T4 and TSH ELISAs are 3.7–300 ng/mL and 49.4–4000 pg/mL, respectively.

### 2.6. Histopathological Evaluation

The pancreas was fixed in 4% paraformaldehyde and embedded in paraffin. Then, it was sectioned at 5 *μ*m thickness and stained with hematoxylin and eosin (H&E) for observation.

### 2.7. Localization of TSHR and Insulin Determined by Immunolabeling

Briefly, the pancreatic tissue sections were deparaffinized, rehydrated, and fixed in 4% paraformaldehyde. Cultured MIN6 cells were fixed using 4% paraformaldehyde. Both samples were permeabilized in 0.3% Triton X-100 for 5 min and then incubated with 5% BSA for 30 min. Anti-insulin (1 : 200) and anti-TSHR (1 : 50) antibodies were applied at 4°C overnight, followed by incubation with secondary antibodies (Invitrogen, Carlsbad, CA) for 30 min. Nuclei were counterstained with DAPI. TSHR and insulin expression was visualized using a confocal microscope (FV10i Fluoview, Olympus, Japan) and fluorescence microscope (IX71, Olympus).

### 2.8. Oral Glucose Tolerance Test (OGTT)

Adult male offspring were fasted overnight for 12 h. Blood samples were obtained from tail cuts to determine blood glucose at time zero. Then, glucose (2 g/kg) was administered orally to mice. At 30, 60, and 120 min after glucose administration, blood glucose was measured by an automatic glucometer (Roche, Germany).

### 2.9. Islet Isolation and Glucose-Stimulated Insulin Secretion (GSIS) Assay

Mice were anesthetized by an intraperitoneal injection of pentobarbital sodium (60 mg/kg), and the abdomen was exposed. Ice-cold HBSS (5 mL, pH 7.6, and 0.037 mM containing CaCl_2_) with 1 mg/mL collagenase V was injected through the bile duct, and then the swollen pancreas was removed and digested at 37°C for 27–29 min. Then, the pancreas was shaken vigorously, and the digestion was terminated by 1 mL FBS in 25 mL ice-cold HBSS. The suspension was filtered to remove any undigested tissue. After washing with HBSS, islets were resuspended with 3 mL HBSS and 1 mL RPMI 1640 medium and handpicked under a stereomicroscope.

Batches of 20 islets were transferred into a 24-well culture plate. The islets were first incubated in 750 *μ*L glucose-free Krebs-Ringer bicarbonate (KRB) buffer for 1 h. Then, the islets were transferred into wells containing 750 *μ*L KRB buffer with 2.8 or 16.7 mM glucose for 1 h. The incubation medium was collected and kept at −80°C until the insulin measurement [11].

### 2.10. Western Blot Analysis

Total proteins were isolated from the mouse pancreas. Briefly, the pancreas was ground in ice-cold extraction buffer, lysed for 30 min, and then centrifuged at 12000 rpm for 30 min at 4°C. The protein concentration was determined by a BCA assay. Proteins were separated by sodium dodecyl sulfate-polyacrylamide gel electrophoresis and transferred onto a polyvinylidene fluoride membrane (Roche, Basel, Switzerland) according to standard procedures. The blots were sequentially incubated with antibodies, including primary rabbit anti-Bcl-2 (1 : 3000), rabbit anti-Bax (1 : 3000), rabbit anti-caspase-3 (1 : 3000), or rabbit anti-GAPDH (1 : 3000) antibodies, and HRP-conjugated secondary antibodies (1 : 5000). Protein bands were visualized using an enhanced chemiluminescence detection kit and recorded on a radiographic film (Alpha Innotech, San Jose, CA). The gray scale value was quantified by ImageJ software.

### 2.11. Statistical Analysis

All calculations and statistical analyses were performed using SPSS for windows version 23.0 (SPSS Inc., Chicago, IL, USA). Data are expressed as the mean ± standard deviation. The Student's *t*-test for independent samples was used to compare the means. *P* < 0.05 was regarded as statistically significant.

## 3. Results

### 3.1. *Tshr*^−/−^ Mice Present with Retarded Somatic and Pancreatic Development

Twenty *Tshr*^+/+^ and 11 *Tshr*^−/−^ male mice were obtained before sacrifice. As shown in Table 2, they were born with similar body weights, but *Tshr*^−/−^ mice exhibited a significantly lower body weight at weaning and adulthood (approximately 82% and 83% of *Tshr*^+/+^ mice, respectively). The ratio of pancreatic to body weights showed no difference between the two groups (*P* > 0.05). *Tshr*^−/−^ mice were fed 100 ppm L-thyroxine powder supplement after weaning and presented with similar serum T4 (*P* > 0.05) and remarkably elevated TSH (approximately 199% of *Tshr*^+/+^ mice) at 8 weeks of age.

### 3.2. TSHR Is Expressed in Pancreatic *β* Cells and *Tshr*^−/−^ Mice Show Atrophy of Islets

**Figure 1:**
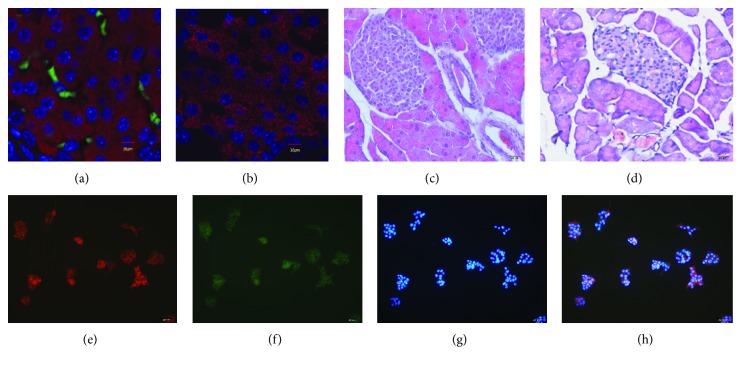
(a, b) TSHR expression in mouse pancreatic islets. (a) TSHR (green) was expressed on the membrane of insulin-secreting cells (red) in the *Tshr*^+/+^ mouse (*n* = 3). (b) No TSHR was detected in *β* cells of the *Tshr*^−/−^ mouse (*n* = 3) as observed by laser scanning confocal microscopy; bar = 30 *μ*m. (c, d) Pancreas microstructures of *Tshr*^+/+^ mice (c, *n* = 3) and *Tshr*^−/−^ mice (d, *n* = 3, stained with H&E, ×400). Three sections were chosen for each animal, and 10 visual fields were observed in each section. (e–h) Double immunofluorescence staining of insulin and TSHR in MIN6 cells. (e) Insulin-immunoreactive MIN6 cells (red). (f) TSHR-immunoreactive MIN6 cells (green). (g) Blue is DAPI counterstaining. (h) Merged images in (e), (f), and (g) show cells double-positive for insulin and TSHR. Bar = 20 *μ*m.

TSHR has been reported to be expressed in extrathyroidal tissues such as pancreatic islets [9]. Pancreatic cells mainly consist of beta cells, which are the major functioning cells in glucose metabolism. However, no available data have directly proved that pancreatic *β* cells express TSHR. Double immunofluorescence staining of TSHR and insulin was performed to precisely locate TSHR expression in *β* cells *in vivo* and *in vitro*. As a result, we confirmed expression of TSHR in mouse pancreatic *β* cells (Figures 1(a) and 1(b)) and MIN6 cells (Figures 1(e)–1(h)).

H&E staining was also performed to investigate the effect of TSHR mutation on pancreatic histology. We observed significant atrophy of the pancreatic acinus and islets, as well as a reduced cell mass, enlarged cell gap, and vacuole-like changes in *Tshr*^−/−^ mice (Figures 1(c) and 1(d)).

### 3.3. TSHR Mutant Mice Exhibit Impaired Glucose Tolerance and Insulin Secretion

**Figure 2:**
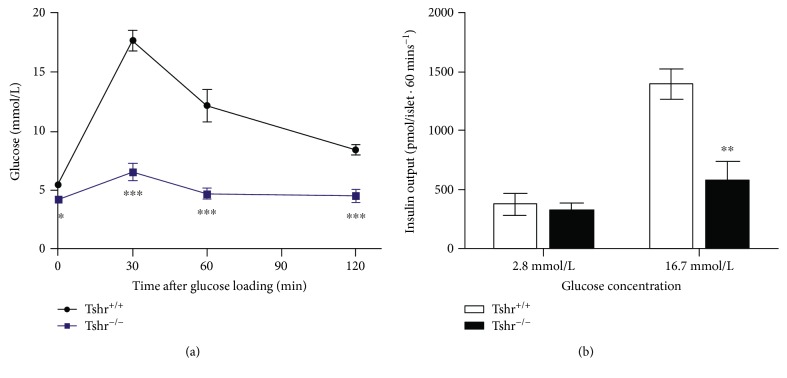
Comparison of OGTT *in vivo* and the GSIS test of islets *in vitro* between the two groups. Ten *Tshr*^+/+^ and five *Tshr*^−/−^ mice were used in these two experiments, and 20 islets were isolated from each mouse for GSIS. Values are shown as means ± S.D. and analyzed by an independent *t*-test. ^∗^*P* < 0.05, ^∗∗^*P* < 0.01, and ^∗∗∗^*P* < 0.005.

An OGTT was performed to evaluate the effect of TSHR deletion on glucose metabolism in adult mice. Compared with *Tshr*^+/+^ mice (*n* = 10), the blood glucose level of *Tshr*^−/−^ mice (*n* = 5) was lower at all time points (*P* < 0.05). The highest blood glucose at 30 min post loading was only 42% of that in wildtype mice (7.84 ± 1.01 mmol/L*vs.*18.79 ± 1.09 mmol/L). The time point of the glucose peak and downward trend of blood glucose were similar in the two groups (Figure 2(a)).

*In vitro*, pancreatic islets were separated and purified to perform a GSIS. Freshly isolated islets were treated with various glucose concentrations to measure the insulin output. Similar insulin secretion was found between the two groups with a low concentration of glucose stimulation. However, insulin output was reduced by 59% in *Tshr*^−/−^ mice under stimulation by high glucose (Figure 2(b)).

### 3.4. TSHR Deletion Is Related to Enhanced Pancreatic Cell Apoptosis

**Figure 3:**
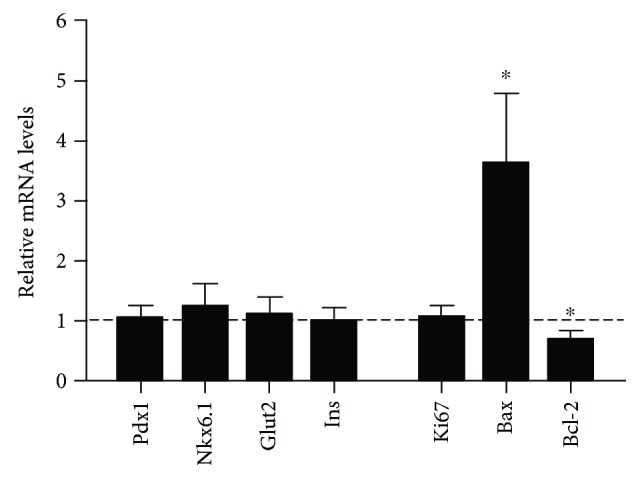
Altered mRNA expression in the mutant mouse pancreas. The mRNA levels of Pdx1, Nkx6.1, Glut2, insulin, Ki67, Bax, and Bcl-2 in *Tshr*^−/−^ mice pancreas were validated by real-time PCR. RNA was extracted from *Tshr*^+/+^ mice (*n* = 7) and *Tshr*^−/−^ mice (*n* = 3).

**Figure 4:**
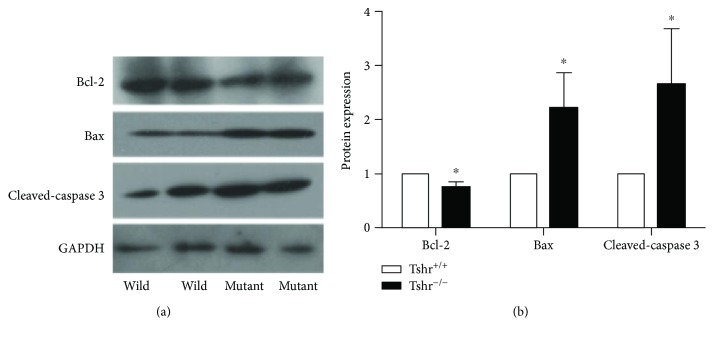
Comparison of apoptosis-related protein expression in the pancreas of the two groups. (a) Protein levels of Bcl-2, Bax, and cleaved caspase 3 in the pancreas were measured by western blotting. (b) Protein expression levels were quantified by ImageJ. GAPDH was used as a loading control. ^∗^*P* < 0.05, compared with the control group. Protein was extracted from *Tshr*^+/+^ mice (*n* = 7) and *Tshr*^−/−^ mice (*n* = 3).

Our mRNA analyses showed that the expression of pancreatic functional markers, such as Pdx1, Nkx6.1, insulin, and Glut2, did not demonstrate significant changes between the two groups (Figure 3). Ki67, a typical cell proliferation marker, showed no difference compared with wildtype mice at the mRNA level after TSHR deletion. However, the modulation of apoptosis-related mRNA expression had remarkable changes in the mutant mouse pancreas. Expression of Bax was remarkably upregulated to 363% compared with *Tshr*^+/+^ mice. Moreover, the mRNA level of Bcl-2 was reduced by 31% in *Tshr*^−/−^ mice. The variation in cell apoptosis-related protein levels of *Tshr*^−/−^ mice is shown in Figure 4. Expression of Bax and cleaved caspase 3 was significantly increased to 220% and 213%, respectively, after TSHR deletion, while the expression of Bcl-2 was remarkably decreased by 25% in *Tshr*^−/−^ mice.

## 4. Discussion

Along with TSHR expression in various tissues, the extrathyroidal effect of TSH is attracting attention. In this study, we confirmed expression of TSHR in pancreatic *β* cells by double immunofluorescence labeling of TSHR and insulin both *in vivo* and *in vitro*. We found that *Tshr*^−/−^ mice exhibited retardation of somatic development after birth and pancreatic islet atrophy in adulthood. Enhanced cell apoptosis, rather than cell dysmaturity or impaired proliferation in the pancreas of *Tshr*^−/−^ mice, was associated with decreased insulin output in response to glucose stimulation.

In animal studies of thyroid dysfunction affecting offspring development, male littermates are often used [12, 13]. Male mice were also chosen in a study by Wang et al. using the same TSHR knockout mouse model [8]. The preference of males may be associated with estrogen-related thyroid pathophysiology [14] and estrous cycle-related hormone changes. Previous studies showed a marked variation in expression of the insulin gene, insulin secretion, and significant fluctuation of thyrotropin-releasing hormone during the estrous cycle [15–17]. Based on the above studies, we chose male offspring for animal experiments.

The thyroid gland of TSHR mutant mice cannot synthesize a sodium iodide symporter. Therefore, thyroid globulin is not able to be iodinated. As a result, *Tshr*^−/−^ mice are unable to produce thyroid hormone, and their survival after weaning depends on thyroid hormone supplementation. In this study, TSHR mutant mice were fed L-thyroxine powder after weaning and exhibited the same T4 level as wildtype mice in adulthood, suggesting that the difference in their phenotype could be excluded by thyroid insufficiency.

TSHR mutation blocks the binding of TSH to its receptor and results in an elevated TSH level in circulation. Studies have shown that TSHR also exists in extrathyroidal tissues such as islets [9], thymus, pituitary, testes, kidneys, brain, heart, and bone [18]. TSHR mutant mice had a smaller pancreas, but a similar pancreas to body weight ratio in the present study, which was coincident with the changes in the liver, heart, and kidneys reported by Wang et al. We suspected that their retarded physical growth was related to TSHR deletion and obstructed TSH modulation throughout the whole body.

OGTT is the most direct method to evaluate glucose metabolism *in vivo*. Our results showed that glucose concentrations at all time points throughout the experiment were significantly lower in TSHR mutant mice. Because the results were consistent with the data reported by Wang et al. using the same animal model, we considered them to be authentic. When treated with a lower concentration of glucose in the GSIS, isolated pancreatic islets produced an equivalent amount of insulin in the two types of mice. The reduced fasting blood glucose observed in TSHR mutant mice may result from decreased hepatic glucose output [8]. Compared with wildtype mice, the insulin level was remarkably lower when challenged by high glucose in *Tshr*^−/−^ mice, indicating damage to *β* cell functions. However, glucose levels after loading did not elevate to a relatively high range as a result of insulin deficiency. Glucose homeostasis is regulated by a network of hormones. In addition to glucose-lowering hormones, there is a series of glucose-elevating hormones such as glucocorticoids, growth hormone, and glucagon. We suspected that the abnormality in low glucose after loading could be affected by the variation in glucose-elevating hormones related to TSHR deletion, which needs further investigation.

In other animal models such as *hyt/hyt* mouse and zebrafish, the influence of TSH-TSHR signaling failure on organ development and functions has only been studied in the thyroid. In *hyt/hyt* mutant mice, thyroid hypoplasia is observed postnatally [19], whereas TSHR mutant zebrafish have a significant reduction in the number and size of functional follicles [20]. To investigate the effect of TSHR deletion on pancreatic functions, we performed further histological and molecular experiments. Histology showed pancreatic injury in adult *Tshr*^−/−^ mutants. The cell number reduction was a remarkable change in the islets, which may be attributed to either obstructed cell proliferation or increased cell apoptosis.

It is generally accepted that Pdx1 is an important insulin promoter activator required for pancreas organogenesis and *β* cell maturation [21, 22]. Nkx6.1 is another transcription factor uniquely expressed in mature *β* cells. It maintains the normal functions of postnatal *β* cells by enhancing cell proliferation and mass expansion [23]. Glut2 acts as a glucose sensor on the membrane of *β* cells, and together with insulin are considered as cell maturation markers. We evaluated the expression of these genes in the adult mouse pancreas at the mRNA level and found no significant differences between the two groups (Figure 3). Moreover, the mRNA level of cell proliferation marker Ki67 did not change in the mutant mouse pancreas. The absence of TSH regulation may presumably have little effect on pancreatic cell proliferation and maturation.

Expression of cell apoptosis-related genes, such as Bax and Bcl-2, was also evaluated in the pancreas. The fold change in mRNA expression of *Tshr*^−/−^ mice to wildtype mice was more predominant for Bax than Bcl-2. Regarded as a cell apoptosis marker, the Bax to Bcl-2 ratio was around three times higher in *Tshr*^−/−^ mice than in wildtype mice. We speculated that there was a higher cell apoptosis level in the pancreas of TSHR mutants. Further experiments showed that the protein levels of Bax and cleaved caspase 3 were coincidently increased in *Tshr*^−/−^ mice (Figure 4). Pancreatic cell apoptosis is enhanced by blockade of TSH-TSHR signaling, which could be a reasonable explanation for the cell loss and function deterioration of *β* cells, because they constitute the majority of pancreatic cells.

In summary, our study revealed TSHR expression in mouse pancreatic *β* cells. TSH has an extrathyroidal modulatory function in *β* cells by binding to its receptor. TSHR deletion was associated with the deterioration of pancreatic functions in male mice, which was mainly manifested as enhanced cell apoptosis. Furthermore, glucose metabolism was disturbed as a result of impaired insulin secretion after loading in TSHR mutant mice.

## Figures and Tables

**Table 1:** Primers for RT-PCR.

Target genes	Primer sequence (5′-3′)		Size (bp)
	Forward	Reverse	
TSHR	CAGGGTGGAGACGCACACTC	AGAGAGTCCCACAACAGTC	590
TSHR mutant	AGAGAGTCCCACAACAGTC	TCCTTGAAGAAGATGGTGCG	173
Bcl2	GGCATCTTCTCCTTCCAG	ATCCCAGCCTCCGTTAT	441
Bax	GTTACAGGGTTTCATCCAGG	CGTGTCCACGTCAGCAAT	183
Pdx1	TGGGCAGGAGGTGCTTACA	TTCCACTTCATGCGACGGTT	206
Nkx6.1	GACAGCAAATCTTCGCCCTG	ACCAGACCTTGACCTGACTC	117
Insulin	CACCCCACCTGGAGACCTTA	TGAAACAATGACCTGCTTGCTG	153
Glut2	GGGACTTGTGCTGCTGGATA	GAACACGTAAGGCCCAAGGA	244
Ki67	TCACCTGGTCACCATCAAGC	TCAATACTCCTTCCAAACAGGCA	87
GAPDH	CATCAAGAAGGTGGTGAAGC	CATCGAAGGTGGAAGAGTGGG	119

**Table 2:** Baseline information of *Tshr*^−/−^ mice.

	*Tshr* ^+/+^ mice *n* = 20	*Tshr* ^−/−^ mice *n* = 11	*P* value
BW at born (g)	1.39 ± 0.06	1.43 ± 0.10	0.708
BW at weaning (g, 3 wk)	7.93 ± 0.38	6.53 ± 0.39	0.037^∗^
BW at adulthood (g, 8 wk)	20.96 ± 0.64	17.36 ± 1.98	0.045^∗^
PW at adulthood (g, 8 wk)	0.39 ± 0.03	0.23 ± 0.04	0.001^∗∗∗^
PW/BW (%)	1.46	1.43	0.82
T4 (ng/mL, 8 wk)	9.59 ± 0.24	10.29 ± 0.38	0.112
TSH (pg/mL, 8 wk)	649.5 ± 41.84	1289.0 ± 100.90	<0.0001^∗∗∗^

Values are shown as means ± S.D. and analyzed by an independent *t*-test. BW, body weight; PW, pancreatic weight; T4, thyroxine; ^∗^*P* < 0.05 and ^∗∗∗^*P* < 0.005.

## Data Availability

All data used to support the findings of this study are available from the corresponding authors upon request. A request for data may be sent through the following e-mail address: doc_kunwang@163.com.
